# Virus stealth technology: Tools to study virus cell-to-cell transmission

**DOI:** 10.1371/journal.ppat.1012590

**Published:** 2024-10-09

**Authors:** Peiqi Yin, Caroline K. Martin, Margaret Kielian

**Affiliations:** Department of Cell Biology, Albert Einstein College of Medicine, Bronx, New York, United States of America; Mount Sinai School of Medicine, UNITED STATES OF AMERICA

## Introduction

To successfully infect a cell, viruses must overcome the host’s antiviral defenses including the adaptive immune response that produces antiviral antibodies. Many viruses have evolved strategies to infect even in the presence of neutralizing antibodies via a process of virus cell-to-cell transmission. These strategies and mechanisms are summarized and reviewed in [1–3]. Here, we briefly review and discuss different experimental approaches to study virus cell-to-cell transmission in vitro and in vivo. We focus here on selected examples of enveloped animal viruses including the serious human pathogens chikungunya virus (CHIKV) and human immunodeficiency virus-1 (HIV-1), but many viruses have been studied for cell-to-cell transmission [1–3].

### What is virus cell-to-cell transmission?

**Fig 1:**
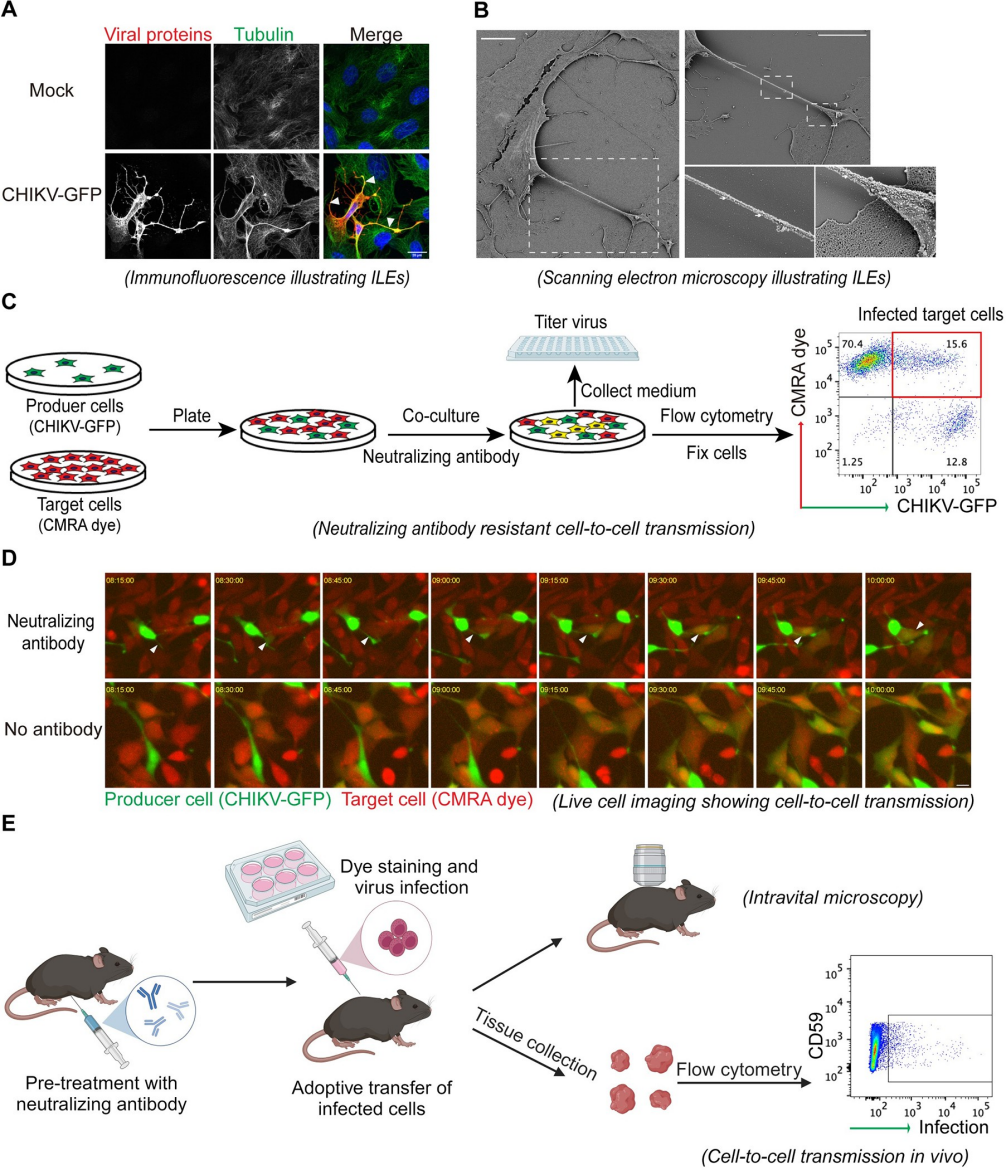
Illustration of approaches for studying virus cell-to-cell transmission in vitro and in vivo. The alphaviruses CHIKV and SINV are used as examples (panel E includes HIV). (A) Immunofluorescence microscopy illustrating ILEs. MEFs were infected with a CHIKV-GFP reporter virus for 11 h, fixed, permeabilized, and stained with antibodies to the viral envelope proteins and tubulin (micrographs are pseudo-colored and the GFP channel is not displayed). Scale bar, 20 μm. Arrowheads point out ILEs. (B) SEM illustrating ILEs. Vero cells were infected with SINV for 9 h, processed for SEM and imaged using a Zeiss Supra 40 field emission SEM. Scale bar, 10 μm. Adapted from [12]. (C) Neutralizing antibody-resistant cell-to-cell transmission. Producer MEFs were infected with CHIKV-GFP; target MEFs were pre-stained with Cell Tracker Orange CMRA dye. Producer and target cells were then co-cultured in the presence of neutralizing antibody against CHIKV for 12 h. The co-culture media were collected to titer free virus particles, and the cells were fixed and analyzed by flow cytometry. Adapted from [13]. (D) Live cell imaging showing cell-to-cell transmission in presence of neutralizing antibody. The co-culture system described in C was incubated with or without neutralizing antibody. Cell contact formation and infection of target cells were monitored by live cell imaging using a DeltaVision Core Microscope. Images show a time range from 8.25–10 h postinfection. Scale bar, 20 μm. Arrows point out ILEs. (E) Cell-to-cell transmission in vivo assayed by flow cytometry and/or intravital imaging. In the CHIKV example, mice were pre-treated with neutralizing antibody before being injected in the footpad with CHIKV-Venus-infected MEFs prestained with CMRA-dye (adoptive transfer). Spread of infection can be assessed either by analysis of ipsilateral ankle tissues by flow cytometry [13] as illustrated for CHIKV or by intravital microscopy to follow infected producer cells (as has been done for HIV-1 [28]). Diagram was created with BioRender.com. CHIKV, chikungunya virus; ILE, intercellular long extension; MEF, mouse embryonic fibroblast; SEM, scanning electron microscopy; SINV, Sindbis virus.

Viruses can spread either by diffusion through the extracellular space (cell-free virus spread), enabling long distance transmission, or by direct cell–cell contacts, promoting rapid, short-range dissemination. During cell-free spread, virions are released into the extracellular space and are thus vulnerable to detection and inhibition by neutralizing antibodies. In contrast, in cell-to-cell spread virus particles (or viral genomes in some cases) are transmitted by contacts between an infected virus “producer” cell and a neighboring “target” cell (e.g., Fig 1A and 1B), which can shield the virus from antibody. For certain viruses, direct cell-to-cell transmission at the contact sites can promote rapid virus dissemination and evasion of anti-viral and immune barriers, thus contributing to viral persistence and latency. Examples of this include Herpes Simplex virus type 1, which uses cell-to-cell transmission to spread directly from mucosal epithelial cells to sensory neurons, and vice versa [4,5]. Cell-to-cell transmission is linked to the establishment of chronic Hepatitis C virus infection in the liver [6,7] and to persistent infection of HIV-1 reservoirs [8]. Direct cell-to-cell spread can also promote resistance to antivirals. For example, transmission of influenza virus through TNTs bypasses inhibition by the neuraminidase inhibitor oseltamivir, which acts to prevent the release of influenza virus particles from host cells [9].

Different viruses can promote formation of a variety of cell–cell contacts to mediate their transmission. For example, transmission can occur via closed-ended structures in which there is no continuity between the cytoplasm of the infected cell and the neighboring cell, such as filopodia-like intercellular long extensions (ILEs) or the tight appositions between cells termed viral synapses. Alternatively, transmission can occur via open-ended, tube-like structures such as tunneling nanotubes (TNTs) [2]. In either case, formation of cell–cell contacts can be accompanied by dramatic, virus-induced changes of the host cell cytoskeleton [3].

### How can cell-to-cell transmission be investigated in vitro?

There is no single standardized assay to study cell-to-cell transmission of animal viruses. Instead, assays are generally virus- and species-specific and depend on reagents such as antibodies and reporter viruses. Each technique also requires specialized expertise and equipment. The methodologies differ, with some assays following virus or viral protein localization without evaluating infection. Other methods depend on preventing cell-free virus spread to allow the independent assessment of infection via cell–cell transmission [3]. A combination of methods gives the most complete picture of transmission and its correlates and requirements.

*Fluorescence and electron microscopy imaging of fixed cells*. In early studies, immunofluorescence (IF) microscopy visualized viral proteins accumulating at cell–cell contact sites, suggesting that these contacts might promote virus cell-to-cell transmission [10]. Since then, imaging methods such as IF microscopy and scanning electron microscopy (SEM) have been instrumental in characterizing and differentiating cellular structures that can mediate virus cell-to-cell transmission, such as ILEs, virological synapses, and TNTs [3,11]. ILEs are formed during infection by several alphaviruses including CHIKV and are actin- and tubulin-positive filopodia-like extensions that form stable contacts with neighboring cells [12,13]. SEM shows virus-sized particles present along the length of the ILEs and at its extensive flattened tip that contacts neighboring cells [12]. HIV-1 infection can induce formation of virological synapses, which are tight adhesive junctions between the infected cell and an uninfected neighbor cell. IF of virological synapses revealed the clustering of receptors on the target cell and viral envelope proteins on the infected cell [3,10] and accumulated HIV particles were observed within these synapses by SEM [14] and electron tomography [15]. Fluorescence in situ hybridization can be used to detect viral genomes at the cell-to-cell contact sites and determine if they colocalize with structural proteins [16,17].

*Live cell imaging*. Live cell imaging can be an effective method for monitoring the spatial and temporal dynamics of transport of fluorescently labeled viruses between cells. Using this technique, particles of the enveloped alphavirus Sindbis virus were observed to localize at the tips of filopodial extensions, from which they were released and internalized by the neighboring cell [12,18,19]. Combined with 3D microscopy, live cell imaging can be used to track cell-to-cell virus spread in relatively thick tissues in vitro. For example, in vitro co-culture of HIV-1–infected T cells with the epithelium in reconstructed mucosa, allowed observation of virus spread across virological synapses by quantitative 3D live cell microscopy [20]. In addition to virus particle dynamics, live cell imaging also allows the study of the cellular changes associated with formation of cell–cell contacts. For example, ILEs formed during alphavirus infection originate from infected cells and form stable contacts with neighboring cells in a process that is accompanied by large-scale cytoskeletal rearrangements [12]. Live cell imaging of this process revealed that ILEs can form either through active outgrowth of the extension, or through a process of retraction, in which the infected cell makes an initial contact with a neighboring cell that is stably maintained as the cells move apart [12]. Combining live cell imaging with fluorescent plasma membrane markers or soluble cytosolic dyes can determine if the contact sites are open-ended and allow transfer of membrane and/or cytosol components between 2 cells [12–21].

*Co-culture assays evaluated by flow cytometry*. To study the relative contributions of cell-free and cell-to-cell transmission to virus infection, it is essential to selectively suppress cell-free infection. This can be achieved by overlaying infected cells with viscous media containing methylcellulose or agarose [3], or with media including virus-neutralizing antibodies. The efficiency of neutralization of cell-free virus can be verified by titering the infectious virus in the culture medium. Co-culturing infected producer cells with target cells in the presence of neutralizing antibody allows characterization of the requirements for cell-to-cell transmission. A fluorescent dye is typically used to distinguish between producer and target cells by pre-labeling one of these cell populations. After co-culture, flow cytometry is used to quantitate the efficiency of cell-to-cell transmission in target cell infection, using antibody staining or viruses expressing a fluorescent reporter to score for infection (Fig 1C) [13]. Such co-culture systems can also be monitored by live cell imaging to directly observe the formation of cell contacts and the resulting infection of target cells (Fig 1D). Co-culture experiments have been instrumental in characterizing the requirements for cell-to-cell transmission of several viruses. For example, CHIKV cell-to-cell transmission was found to require endocytic uptake of virus particles and their exposure to endosomal acidity in the target cells, similar to free virus infection [13]. However, cell-to-cell transmission was independent of the CHIKV receptor MXRA8, presumably reflecting the close association and locally high virus concentration at the interface between the producer and target cells. Some monoclonal antibodies (mAbs) to CHIKV inhibit the formation of stable ILE, independent of mAb neutralizing capability [13]. While such mAbs cannot be used to establish co-culture assays, they have been very useful in demonstrating the role of ILE-mediated contacts in CHIKV cell-to-cell transmission. Alternatively, shaking of cell cultures or seeding of cells in transwells can also be used to prevent the formation of stable cell-to-cell contacts [22,23]. By comparing infection in static versus shaking culture systems, mathematical modeling was used to estimate the contribution of cell-to-cell transmission to HIV-1 spread in vitro [24,25].

### How can cell-to-cell transmission be investigated in vivo?

While in vitro assays are very useful to study specific aspects of cell-to-cell transmission, they do not necessarily emulate natural virus infection in vivo. The existence and/or relative contribution of cell-to-cell transmission to viral infection in vivo has therefore been debated. To directly address this, the in vivo approaches summarized below allow the study of cell-to-cell transmission during infection of a model organism. Such in vivo approaches can make it possible to address the role of cell-to-cell transmission in persistent infection and in evasion of neutralizing antibody responses.

*Adaptive transfer of producer cells*. To study this, mice are prophylactically treated with neutralizing antibodies and then inoculated either by injection of free virus, or by adoptive transfer of virus-infected producer cells. This type of system has been tested for several viruses including HIV and CHIKV [13,26]. In each case, free virus infection was neutralized by antibody treatment, while adoptive transfer resulted in infection of endogenous cells, suggesting cell-to-cell virus transmission (Fig 1E). Such HIV cell-to-cell transmission promoted multicopy infection in an acute transmission model based on humanized mice [26].

At present, infection by adoptive transfer is a rather complex and time-consuming process. It is possible that alternative systems can circumvent the need for adoptive transfer to allow evaluation of cell-to-cell transmission in vivo. For example, mice could be inoculated with cell-free virus and cell-to-cell contacts inhibited by specific anti-viral antibodies [13], with a potent neutralizing mAb used to completely block cell-free virus infection. If cell-to-cell transmission is responsible for neutralizing Ab-resistant infection, it should be decreased by specific inhibition of cell-to-cell contacts. Identification of specific tools such as anti-viral antibodies is essential to develop novel animal models to study cell-to-cell transmission and to determine its possible contribution to disease and viral persistence in vivo. Independent of the method, thorough characterization of the tools and careful interpretation of the data remain essential.

*Intravital imaging of virus spread*. While virus-transmitting cell-to-cell contacts can readily be visualized in vitro, observation of cell-to-cell transmission in vivo relies on more complex techniques such as multi-photon intravital microscopy. Surgical exposure of the infected tissue in the live animal allows for imaging depths of up to 1 mm and provides dynamic and single-cell-resolved information [27]. Using this approach, anchored HIV-1-infected T cells were observed to form stable contacts with uninfected CD4 lymph node cells in humanized mice, suggesting the formation of virological synapses in vivo [28]. Together with the observation that HIV-1 infection spreads in clusters within the splenic lymphoid tissue of humanized mice, this provides evidence for direct cell-to-cell transmission in vivo [26]. Although these infected cell clusters suggest that HIV-1 spread is limited to areas surrounding infected cells, studies including neutralizing Abs could provide additional evidence of the relative contributions of cell-free versus direct cell-to-cell virus spread.

### Future question: Can inhibition of cell-to-cell transmission serve as an antiviral strategy?

While much remains to be learned, the potential roles of cell-to-cell transmission in viral immune evasion and persistence could make it an important focus of antiviral strategies. For example, inhibition of the various cell contacts involved would block virus transmission by the cell-to-cell route. One mechanism of inhibition could take advantage of blocking Abs, as in the example of the specific Abs to the CHIKV envelope proteins that inhibit cell-to-cell contact formation and transmission [13]. The concept of Ab-mediated inhibition of cell-to-cell transmission might extrapolate to other virus infections including during human infections and could also be considered as part of vaccine development. Another approach would be to develop pharmacological inhibitors of cell-to-cell transmission. Dramatic cytoskeletal remodeling of host cells occurs during the formation of the specialized contacts that mediate cell-to-cell transmission. While specific targeting of the relevant cellular signaling and/or host factors that promote remodeling may be challenging, it could also provide an important tool to block viral cell-to-cell transmission. Thus, development of antibodies and inhibitors is not only central to the study of cell-to-cell transmission in viral pathogenesis, it might also inform future anti-viral treatments for human infections.
